# Differentiation of malignant brain tumor types using intratumoral and peritumoral radiomic features

**DOI:** 10.3389/fonc.2022.848846

**Published:** 2022-07-28

**Authors:** Dongming Liu, Jiu Chen, Honglin Ge, Xinhua Hu, Kun Yang, Yong Liu, Guanjie Hu, Bei Luo, Zhen Yan, Kun Song, Chaoyong Xiao, Yuanjie Zou, Wenbin Zhang, Hongyi Liu

**Affiliations:** ^1^ Department of Neurosurgery, The Affiliated Brain Hospital of Nanjing Medical University, Nanjing, China; ^2^ Institute of Neuropsychiatry, The Affiliated Brain Hospital of Nanjing Medical University, Fourth Clinical College of Nanjing Medical University, Nanjing, China; ^3^ Department of Neurosurgery, Institute of Brain Sciences, The Affiliated Brain Hospital of Nanjing Medical University, Nanjing, China; ^4^ Department of Pathology, The Affiliated Brain Hospital of Nanjing Medical University, Nanjing, China; ^5^ Department of Radiology, The Affiliated Brain Hospital of Nanjing Medical University, Nanjing, China

**Keywords:** radiomics, glioblastoma, lymphoma, brain metastases, peritumoral regions

## Abstract

Tumor infiltration of central nervous system (CNS) malignant tumors may extend beyond visible contrast enhancement. This study explored tumor habitat characteristics in the intratumoral and peritumoral regions to distinguish common malignant brain tumors such as glioblastoma, primary central nervous system lymphoma, and brain metastases. The preoperative MRI data of 200 patients with solitary malignant brain tumors were included from two datasets for training. Quantitative radiomic features from the intratumoral and peritumoral regions were extracted for model training. The performance of the model was evaluated using data (*n* = 50) from the third clinical center. When combining the intratumoral and peritumoral features, the Adaboost model achieved the best area under the curve (AUC) of 0.91 and accuracy of 76.9% in the test cohort. Based on the optimal features and classifier, the model in the binary classification diagnosis achieves AUC of 0.98 (glioblastoma and lymphoma), 0.86 (lymphoma and metastases), and 0.70 (glioblastoma and metastases) in the test cohort, respectively. In conclusion, quantitative features from non-enhanced peritumoral regions (especially features from the 10-mm margin around the tumor) can provide additional information for the characterization of regional tumoral heterogeneity, which may offer potential value for future individualized assessment of patients with CNS tumors.

## 1 Introduction

Malignant tumors of the central nervous system (CNS) are among the most resource-consuming and disabling diseases in neurology. Generally, typical neuroradiological findings in conventional magnetic resonance imaging (MRI) raise the initial suspicion for CNS tumors. Radiologists rely upon visible characteristics to describe CNS tumors, with contrast-enhanced regions being important elements for evaluation. In recent years, quantitative parametric MRI techniques have further facilitated substantial progress in the diagnosis, therapy monitoring, and evaluation of CNS tumors (1). More recently, by extracting high-throughput features, radiomics technologies further allow the non-invasive capture of microscale tumoral information (2, 3). At present, computer-assisted medical diagnosis technology has been successfully applied to clinical diagnosis (4), molecular marker evaluation (5), and prognosis prediction (6) of CNS tumors. However, due to overreliance on the visible regions of the tumor habitat, such as the necrosis, enhancement, and edema parts, many studies seem to mainly focus on the intratumoral information, ignoring the peritumoral brain zone (PBZ) that also plays a prominent role in the progression and recurrence of malignant CNS tumor (7–9).

Currently, the volume of enhancement in contrast-enhanced MRI (CE-MRI) usually serves as a radiological reference for defining the burden of high-grade CNS tumors, which guides invasive biopsy or surgical resections (10) as well as radiation therapy planning. Due to their infiltrative and heterogeneous nature, GBMs are difficult to evaluate and treat (7, 11), and complete resection of the contrast-enhanced parts is considered radical resection (10). In cases with complete radical resection of the enhanced portion of GBM, however, 85% of recurrences are still localized to the resection PBZ margin (9), which ultimately leads to treatment failure. Previous studies have shown that GBM cells can be located a few centimeters away from the tumor enhancement margin in CE-MRI (7, 8, 12), suggesting that the radiological boundary of the tumor does not exactly match the cytological boundary. The glioma infiltration may be unenhanced before the formation of high-permeability neovascularization (8, 13). Therefore, no enhancement does not suggest the absence of tumoral infiltration in PBZ. The hyper-focusing on the enhanced portions of the tumor in CE-MRI effectively guides the surgical target, which may also lead to the neglect of residual non-enhancing parenchyma in PBZ regions in clinical practice or radiological research.

In the context of CNS tumor imaging, the information provided by T2-weighted sequences (T2) is generally related to tissue water content. The coverage areas of edema in T2 caused by brain tumors are often larger than the enhancement lesion itself and are often considered to represent the peritumoral area in many radiomics studies (14, 15). Nevertheless, just like the spatio-temporal heterogeneity of GBMs at the molecular scale (16), the GBMs similarly have heterogeneous phenotypes at the macroscopic radiological scale (17). The edema of the tumor is variable and affected by different factors, which may not reflect the real condition of peritumoral invasion. Remarkably, based on the tissues even from similar edema regions of different GBMs, the content of tumor cells confirmed by biopsy can be highly variable (10–80%) (17), suggesting that using only hyperintensity in T2 (edema) to assess the characteristic of PBZ may be challenging. Thus, can the distance-quantitative peritumoral features of the tumor be used to weaken the effect of tumor radiological heterogeneity in different MRI data? Whether the quantitative features around the PBZ can better capture the characteristics of a malignant CNS tumor is currently unknown.

Among malignant CNS tumors, preoperative differentiation of GBM, brain metastasis (META), and primary central nervous system lymphoma (LMPA) can be challenging. The three types of tumors can present similar enhancement and edema patterns on traditional MRI, resulting in difficulties in clinical differentiation before surgery. The therapeutic strategies for these tumors are entirely different: newly diagnosed GBM often requires maximum resection, followed by chemotherapy and radiotherapy (18); META requires postoperative stereotactic radiosurgery and systemic treatment for the primary tumor (19), while LMPA usually only requires stereotactic biopsy followed by chemotherapy and sometimes combined with whole-brain radiation therapy (20). Therefore, preoperative accurate identification among these tumors has a significant clinical relevance. Onishi et al. (21) have attempted to discriminate the tumors with several perfusion indicators. The small sample size and single-center data limit the generalizability of the findings. Additionally, several recent radiomics studies have tried to perform differential diagnoses between GBM, META, and LMPA (4, 22, 23). However, on one hand, most radiomics studies only focus on the binary classification problems of the tumors mentioned, which limits the generalization and clinical application of the models. On the other hand, these studies regarded almost exclusively intratumoral features and edema itself, leading to the loss of peritumoral information. In this exploratory and multicenter study, we not only evaluated the visible intratumoral regions of interest (ROI) but also focused on the distance-quantitative peritumoral regions from PBZ. In doing so, we attempted to address two questions in this study. First, we examined whether radiomic analysis will facilitate the multiple classifications of the three common malignant CNS tumors. Second, we explored if the features extracted from PBZ regions can provide additional biological information for the evaluation of a malignant brain tumor. Additionally, binary classifications (GBM-LMPA, GBM-META, and LMPA-META) were also performed using the optimal features.

## 2 Materials and methods

### 2.1 Patient enrollment

**Figure 1:**
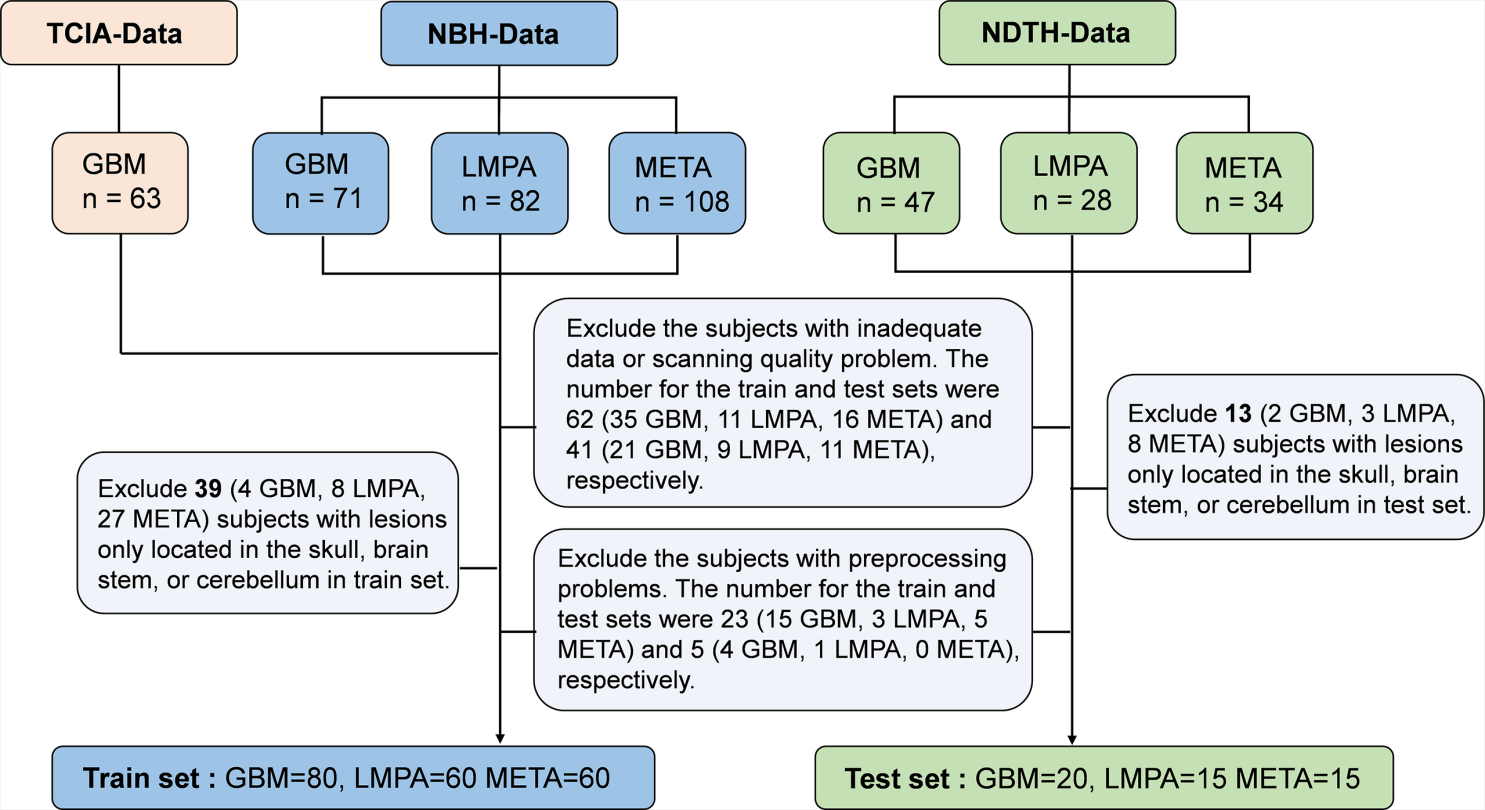
Data organization of the patients from three data sets.

Patients diagnosed with malignant CNS tumors were recruited from three cohorts. Retrospective MRI data (GBM, META, and LMPA) were collected from the Nanjing Brain Hospital-Brain Tumor Neuroimaging Project database (from January 2016 to June 2020) for training, which is described in **Supplementary Materials S1**. Additionally, half of the GBM data in the training cohort was also included from The Cancer Imaging Archive project (TCIA, http://www.cancerimagingarchive.net). In order to assess the generalization of the model, three types of patients in the test set were included from the Nanjing Drum Tower Hospital (from July 2019 to December 2020) in the same ratio as that in the training group. For the training set, 324 patients with pathologically confirmed malignant brain tumors were originally included, including 134 patients with GBM (63 patients were enrolled from the TCIA database), 82 patients with LMPA, and 108 patients with META. For the testing set, 109 patients were originally included (*n* = 47 for GBM, *n* = 28 for LMPA, and *n* = 34 for META). The exclusion criteria were as follows: (1) patients with inadequate MRI data or scanning quality problems, (2) patients with a lesion only located in the skull, brain stem, or cerebellum, (3) patients with MRI data that have preprocessing problems. The enrollment flow chart is briefly illustrated in **Figure 1**. This study was approved by the Institutional Ethical Committee for Clinical Research of the Affiliated Brain Hospital of Nanjing Medical University.

### 2.2 Data acquisition and pre-processing

The details about the sequence parameters of the T1CE and T2 protocols are described in **Supplementary Materials S1**. The MRI scanning parameters varied among the different centers, reflecting the heterogeneity of imaging data in the clinical context. Further description of data pre-processing and quality control is provided in **Supplementary Materials S2**.

### 2.3 Region-of-interest segmentation

**Figure 2:**
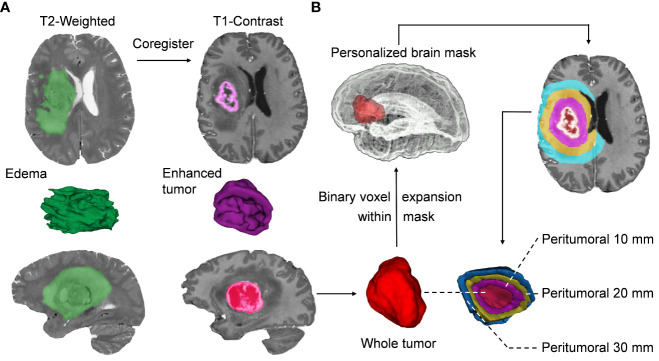
Segmentation of the intratumoral and peritumoral subregions. **(A)** After image preprocessing, the visible tumors were segmented into different subregions, including enhancement, whole tumor, and edema, based on a semi-automatic threshold and seed-growing algorithm. **(B)** The same approach was used to segment individualized brain masks without ventricle, cortical sulci, and infratentorial structures. The morphological binary expansion of the whole tumor is performed to delineate the peritumoral brain zone (PBZ) regions, which are confined within the individualized whole-brain mask. In each iteration of voxel dilation, a 1-mm^3^ ring around the enhanced lesions will be obtained, and the iterations eventually reach a radial distance of 30 mm. Then, the distance quantitative PBZ regions of interest (ROIs) are obtained by subtraction calculation of the tumor mask. The purple, yellow, and blue ROIs represent the generated ROIs at 10, 20, and 30 mm from the border of the enhanced tumor, respectively.

Radiologically, each tumoral habitat contains two portions, including the traditional subregions and distance-quantitative PBZ subregions. In our study, the traditional subregions refer to ROIs visible to the naked eye on the different MRI sequences, including enhancement and whole tumor parts in T1CE as well as the edema in T2 sequences. The distance-quantitative subregions start from the boundaries of the enhanced lesion in the T1CE sequence and are quantitatively defined by a certain spatial distance, such as 10, 20, and 30 mm. The features extracted from the traditional subregions mentioned above were defined as traditional features. Similarly, features extracted from the distance-quantitative PBZ subregions were defined as quantitative features. The choice of considering 30-mm peritumoral areas was motivated by the fact that the invasion of GBM can reach 3 cm beyond the enhancement boundaries in MRI sequences (7, 8), and this distance also covers the high-frequency recurrence regions (9). For patients with multiple lesions, if the distance between the enhanced borders of the lesions is greater than 5 cm, the largest lesion will be used for delineation, else the tumors would be regarded as a whole for delineation. We used semi-automated and automated methods to segment the traditional regions and PBZ subregions, respectively. The segmentation processes are described in **Figure 2**. Further details are provided in **Supplementary Materials S3**.

### 2.4 Radiomic feature extraction from each subregion

**Figure 3:**
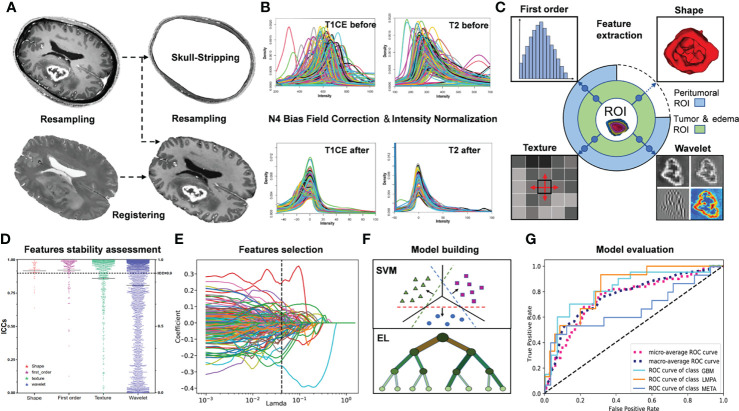
Flow chart describing the present radiomics study. **(A)** Image preprocessing, including skull stripping, resampling, and registration. **(B)** Bias field correction and intensity normalization. **(C)** Based on the quantitative regions of interest, radiomic features were extracted, including shape, first order, textural, and wavelet features from T1CE and T2 images separately. **(D)** Radiomic feature assessment. **(E)** Feature selection using different methods, such as lasso regression or random forest. **(F, G)** Modeling and evaluation using different classifiers, including support vector machine and several ensemble learning methods.

The radiomic features were extracted from the ROIs on T1CE and T2 images using PyRadiomics 3.0 (http://www.radiomics.io/pyradiomics.html) (24). When extracting features in T1CE, ROIs including enhanced tumor (ET) mask, whole tumor (WT) mask, and peritumoral 10/20/30-mm mask in PBZ (P1/P2/P3) were used, respectively. Edema (ED), WT, and P1/P2/P3 masks were likewise used for T2. For each morphologically visible mask (ET, WT, and ED), 14 shapes, 18 first-order, 24 gray-level co-occurrence matrix, 16 gray-level run-length matrix, 16 gray-level size zone matrix, and 14 gray-level dependence matrix as well as 5 neighborhood gray-tone difference matrix were extracted from the corresponding images. We also employed a widely used wavelet filter to explore the potential radiological characteristics of tumors in the wavelet-transformed images. Thus, 744 wavelet features were extracted from decomposed images by a wavelet filter, constituting a total of 851 radiomic features for each ROI in one sequence. The peritumoral ROI was not used to extract the 14 shape features. Finally, a total of 8,412 features (851 × 3 + 837 × 7) were obtained for each tumor. Both sets of ROI generated by the two neuroradiologists would undergo the feature extraction process and result in two sets of radiomic features. A schematic workflow of data pre-processing and radiomic feature extraction is shown in **Figure 3**. A detailed description of these procedures is included in **Supplementary Materials S4**.

### 2.5 Radiomic feature assessment and selection

To evaluate interobserver reproducibility, the intraclass correlation coefficients (ICCs) of the two sets of features were calculated. As suggested by the Image Biomarker Standardization Initiative (25) and Koo et al. (26), features with ICCs greater than 0.9 are considered to be of excellent reliability and would be used for the following analysis. Further descriptions can be found in **Supplementary Materials S4.2**. According to the thumb rule, an effective sample size is needed to cover 10–15 observations of each predictor variable to yield a stable estimation (27, 28). In our study, the training data set included 200 patients, and the maximum number of radiomic features included was 20. Several typical feature selection methods were used, including least absolute shrinkage and selection operator (LASSO), random forest (RF), adaptive boosting (Adaboost), and gradient boosting decision tree (GBDT) as well as extremely randomized trees (ExtraTree). LASSO regression is a robust supervised learning approach that will facilitate variable selection for the high-dimensional dataset. Other algorithms were chosen because of their stable ability for feature importance evaluation. For each method, all features can be sorted based on their feature coefficient or importance, and the top 20 ranked features are used for the corresponding modeling.

### 2.6 Research design and classifier selection

For the same set of features, different feature selection methods and classifiers can present different performances (4). We applied several classifiers including support vector machine (SVM) with linear or radial basis function kernels, RF, Adaboost, GBDT, and ExtraTree classifier. First, we implemented a *K*-fold cross-validation with *K* = 10 to train the models separately using different feature combinations. In order to better evaluate the contribution of the quantitative features, we applied the following four-step modeling strategy. In the first step, the top 20 ranked features of the 2,321 traditional features were used to identify the best-performing model in the triple classification tasks. Then, in step 2, the top 20 features of 3,533 quantitative features were used to train with the best combination identified in step 1. Similarly, in step 3, top features selected from all (2,321 + 3,533) features were also used to fit the model for triple classification tasks. Finally, the best features selected from step 3 are used for binary classifications (GBM-LMPA, GBM-META, and LMPA-META). At each step of the above-mentioned processes, the importance of the features in the corresponding model was calculated. Grid search was used to identify the best parameter combinations in different feature-classifier combinations. Accuracy and AUC were calculated for each combination. A schematic workflow of modeling is shown in **Supplementary Materials S5**.

### 2.7 Statistical analysis

Two-group and multi-group comparisons were assessed with the Mann–Whitney *U*-test and Kruskal–Wallis test. Chi-square tests were used to evaluate the baseline characteristics across groups (gender and tumor hemisphere distribution). The DeLong test was used to evaluate the AUC difference between different models. *P*-values <0.05 were considered statistically significant. The radiomic image processing, statistical analysis, and figure plots were performed using Matlab (version R2013b), Python (version 3.7.4), SPSS (version 21.0, IBM), and R (version 4.0.2) software. Machine learning model training and testing were performed using the scikit-learn library (v0.24.1, https://scikit-learn.org/stable/) implemented in Python.

## 3 Results

### 3.1 Clinical and imaging characteristics

Eventually, 200 patients (GBM = 80, LMPA = 60, and META = 60) and 50 patients (GBM = 20, LMPA = 15, and META = 15) were included in the training and testing cohorts, respectively. The clinical characteristics of the patients in the training and testing sets are listed in **Table 1**. The histogram density curves of raw MRI and preprocessed signal intensities are plotted in **Figure 3B**, and the details are provided in **Supplementary Materials S2**. After preprocessing, all subjects were used for the subsequent radiomic analysis. The proportions of patients with multiple supratentorial lesions (with a distance greater than 5 cm) in the training group and the test group are 6% (two GBM, six LMPA, and four META) and 10% (one GBM, four LMPA, and zero META), respectively.

**Table 1:** The clinical characteristics of patients with central nervous system tumors in the training and testing sets.

	Training set				Testing set			
	GBM (*n* = 80)	LMPA (*n* = 60)	META (*n* = 60)	*p*	GBM (*n* = 20)	LMPA (*n* = 15)	META (*n* = 15)	*p*
Ages, median (range)	60.0 (21–80)	66.0 (31–80)	62.0 (54–76)	0.025^a^	58.0 (35–72)	58.0 (45–79)	59.0 (39–72)	0.865
Gender (male/female)	56/24	35/25	40/20	0.347	11/9	9/6	12/3	0.290
Hemisphere (left/right)	40/40	27/33	36/24	0.244	11/9	9/6	3/12	0.052
Tumor center location and the involved brain area (*N*, number of subjects)								
Tumor cross midline	13	24	4	0.000^a^	2	8	2	0.012^a^
Frontal lobe	27	23	16	0.394	4	5	4	0.671
Temporal lobe	35	16	14	0.020^a^	8	2	0	0.010^a^
Parietal lobe	17	8	14	0.338	7	2	7	0.138
Occipital lobe	12	9	20	0.013^a^	5	3	3	0.916
Insular lobe	6	13	2	0.002^a^	3	4	0	0.108
Other locations	4	13	3		1	9	1	

Age was expressed as the median (range). Age differences between different groups were analyzed using the Kruskal–Wallis rank-sum test. One tumor can involve multiple brain regions at the same time. Chi-square test was used to evaluate the baseline characteristics across groups (gender and tumor hemisphere distribution).

a p-values <0.05 were considered statistically significant.

### 3.2 ROI segmentation and qualitative feature analysis

Based on the normalized images, semi-automatic delineation can be effectively performed using voxel-level seeds and threshold. All patients (*n* = 250) received lesion segmentation that was separately performed by two neuroradiologists. For patients (*n* = 17) with multiple lesions, only the largest lesion was used for segmentation and expansion calculation. For the training set, the stability was observed for shape (ICC = 0.91 ± 0.77), first-order (ICC = 0.92 ± 0.12), texture (ICC = 0.86 ± 0.20), and wavelet (ICC = 0.81 ± 0.32), respectively. These results reflect the good stability of the semi-automated segmentation method. In total, 5,854 of the 8,412 (69.6%) radiomic features showed excellent stability with ICC >0.9, including 31 shape features, 146 first-order features, 484 texture features, and 5,193 wavelet features. The boxplot of ICCs of the radiomic features extracted from four feature classes is provided in **Supplementary Materials S4.2**. For the testing set, the stability of the features was observed for shape (ICC = 0.92 ± 0.64), first-order (ICC = 0.91 ± 0.31), texture (ICC = 0.84 ± 0.31), and wavelet (ICC = 0.83 ± 0.37), respectively.

### 3.3 Optimistic three-classifying model

**Figure 4:**
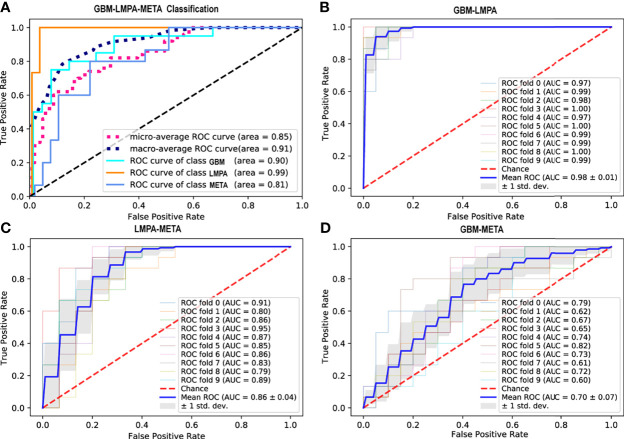
Receiver operating characteristic (ROC) curves for different classification tasks. **(A)** ROC curve of the Adaboost classifier for the differentiation of glioblastoma (GBM), metastases (META), and primary central nervous system lymphoma (LMPA) (using the top 20 features from the traditional and quantitative peritumoral regions). The two dashed lines represent the micro-average and macro-average ROC curves, respectively. **(B–D)** ROC curve of the best classifier for the binary classification between GBM and LMPA, LMPA and META, as well as GBM and META, respectively. Each solid line represents the ROC curve of each fold cross-validation. The thick blue line represents the mean ROC curve (with the standard deviation represented by the gray shadow).

Six classifiers [Lasso_SVM (RBF and linear), Adaboost, RF, GBDT, and ExtraTree] were trained by the training data and then applied to the independent test set to evaluate the performance. Using the top 20 traditional features, the performance of each classifier and the importance of the features are summarized in **Supplementary Materials S6**. Based on intratumoral and edema features, the Adaboost achieved the best performance in the three-classification task (AUC = 0.79 and accuracy = 70.6%). This classifier was selected as the optimal model for the subsequent experiments. While using quantitative features only, the model achieves AUC of 0.765 and accuracy of 62.0%. Once all features (2,321 traditional and 3,533 quantitative features) were put together, using the top 20 features, the Adaboost achieved the highest AUC of 0.91 (**Figure 4A**) and the best mean accuracy value of 76.9%.

### 3.4 The importance and habitat characteristics of the top features

**Figure 5:**
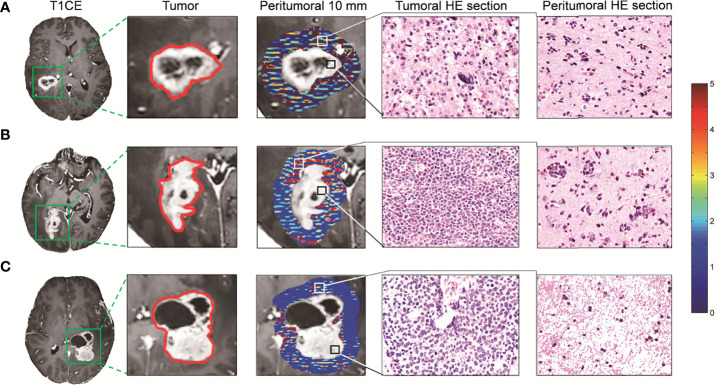
Peritumoral radiological and pathological characteristics of the tumors. Each row represents a single patient. The first column shows the axial slice of patients with central nervous system tumors, and the second and third columns show the enhanced tumor and peritumoral 10-mm regions, respectively. The red outline in the second column is the border of the enhanced tumor. The third column shows the enhanced (black box) tumor and non-enhancing (white box) biopsy regions. The colored heat map represents the expression of wavelet feature around peritumoral 10-mm regions. The last two columns show the pathological section (HE staining; ×400) of the tumor and peritumoral tissue, respectively. **(A)** A female patient with right temporal glioblastoma (GBM); the histopathologic slides of enhanced and peritumoral tissue samples show both biopsy samples infiltrated with malignant GBM cells. **(B)** A male patient with right parietal–occipital primary central nervous system lymphoma; the histopathological sections show the large size of the lymphoid infiltrate in both enhancement and peritumoral biopsy specimens in this patient. **(C)** A female patient with left temporal metastatic adenocarcinoma; the histopathological sections show large numbers of malignant cells which infiltrated the enhanced region, while only a few tumor cells infiltrated the peritumoral 10-mm regions.

Compared to using traditional features only, the Adaboost achieved the best performance when combined with quantitative peritumoral features. The top 20 features showed that the high weights were derived from different tumor habitats. Eleven features were derived from the contrast enhancement (ET, *n* = 4) and whole tumor (WT, *n* = 7) regions inside the tumor in the T1CE sequence. Moreover, five features were picked from the edema regions (ED, *n* = 5) using the T2 sequence. The remaining features were selected from the quantitative regions (*n* = 4). Notably, the whole-tumor ROIs were outlined through the T1CE sequence. Based on these ROIs, the two wavelet features extracted from the T2 sequence (**Supplementary Figure S6**) perform well in the final model, with average importance of 0.065 and 0.037, respectively. The weights of the features in the model are shown in **Supplementary Figure S7**. The features from the peritumoral 10-mm region (**Figure 5**), no matter on T1CE or T2 sequence, likewise have a good contribution to the optimal model. The average weight of the peritumoral features was 0.080 and 0.033, respectively. When focusing on peritumoral features, only two features from 10 mm around the enhanced boundary were ranked at the top and selected for modeling. The Kruskal–Wallis test indicated significant differences among the three groups in the peritumoral 10-mm T1CE-derived wavelet feature (*H* = 6.206, *p* = 0.045). The *post-hoc* analyses revealed significant differences between LMPA and META (*Z* = 2.486, *p* = 0.013). No significant differences were found between GBM and LMBA (*Z* = 1.07, *p* = 0.284) or GBM and META (*Z* = 1.550, *p* = 0.121). No significant differences were observed in the peritumoral 10-mm T2-derived wavelet feature (*H* = 2.653, *p* = 0.265). The weights and statistics details of the final 20 features are summarized in **Supplementary Materials S7**.

### 3.5 Binary-classifying modeling

Based on the best model and the best features determined by the triple classification tasks, binary classifications (GBM-LMPA, GBM-META, and LMPA-META) were also performed. The results of the 10-fold cross-validated ROC curve of the Adaboost classifier in the test cohort are shown in **Figures 4B–D**. The Adaboost model with combined traditional and quantitative features performs better in the task of discriminating between GBM and LMPA (AUC = 0.98, 95% CI: 0.80 to 0.99, accuracy = 89.71%, sensitivity = 87%, and specificity = 100%) (**Figure 4B**) as well as LMPA and META (AUC = 0.86, 95% CI: 0.52 to 0.94, accuracy = 80.33%, sensitivity = 93%, and specificity = 73%) (**Figure 4C**). However, the same model did not perform well when used to discriminate between GBM and META (AUC = 0.70, 95% CI: 0.61 to 0.92, accuracy = 61.71%, sensitivity = 61%, and specificity = 75%) (**Figure 4D**). The weight of the wavelet feature from the peritumoral 10 mm in T1CE in the above-mentioned classification was 0.098, 0.047, and 0.067, respectively. The weights of the features in binary classification are summarized in **Supplementary Materials S8**. Compared to the model that only used traditional features, the model combining traditional and peritumoral features exhibited higher AUC for the binary classification between GBM and LMPA (DeLong test *Z* = 2.24, *p* = 0.025). We also observed an improvement in AUC for the binary classification between LMPA and META, although the difference was not significant (*Z* = 1.69, *p* = 0.090). The peritumoral feature did not provide a significant improvement over the differentiation between GBM and META (*Z* = 0.77, *P* = 0.443).

## 4 Discussion

The diagnosis of CNS malignant tumors may be very challenging to make due to similar imaging findings on conventional MRI. In recent years, machine learning models have begun to outperform previous methods and are gaining increasing attention in differential diagnosis research (4, 23). However, although those models performed well in the validation group, the single-center and dichotomy nature of the studies may limit the generalization of the findings. Based on the fact that radiomic techniques can provide insight into biological information that is invisible to the naked eye (2), relying only on the tumor morphology in a certain sequence may lead to the loss of a lot of important information. Previous studies have tried to divide the tumor habitat into different subregions (6, 29), including necrosis, enhancement, and edema. For different CNS malignant tumors, the tumoral behavior adjacent to the brain parenchyma is different, and peritumoral evaluation may provide extra-biological information (21, 30, 31), especially for differentiating META from other tumors in this study. Compared to the model using only features derived from intratumoral and edema regions, the addition of peritumoral features could improve the performance of the models. These results show that the peritumoral features may contain important biological information that can be uncovered by radiomic analysis. Like the layer of spatial–temporal complexity that peritumoral residual cells have added to tumor biology (7, 30), peritumoral radiomic features may similarly add to the biological imaging of a CNS tumor. Therefore, a better understanding of the tumor behavior also requires a careful assessment of peritumoral infiltration.

Radiologically, the biological information contained in the peritumoral regions has also been verified in other tumors (32, 33). In our study, the distance-quantitative subregions, including 10, 20, and 30 mm from the enhanced margins, were taken into account. According to importance, two features extracted from the 10-mm PBZ regions were selected, which were derived from T1 and T2 sequences, respectively. Compared with using only intratumoral features, the association of the traditional and quantitative features has improved the performance of the model. Our study is congruent with a recent study by Joo et al. (32), such that the radiomic model combining peritumoral features can help to predict brain invasion by meningioma. What is noteworthy is that the top-ranked radiomic features used in their model were derived from the 10-mm-thick brain-to-tumor-interface ROIs. These results altogether indicate that peritumoral radiomic features have the potential to reduce the impact of radiological heterogeneity, enabling quantitative and objective measurements of tumor infiltration. Interestingly, among all the peritumoral features extracted from PBZ regions for final modeling, only the features from peritumoral 10-mm PBZ showed higher weights instead of the ROIs from 20- or 30-mm peritumoral regions. One possible explanation is that, as the distance increases, the distribution of tumor cells becomes sparse. Although tumor infiltration can reach a distance of 3 cm or more, it is difficult to capture long-distance biological information by neuroimaging.

In the present study, most of the radiomic features included in the optimal model were textural features from wavelet-filtered T1CE and T2 images. The texture is a representation of pixel intensity, their inter-relationship, and their distribution, which may or may not be distinguishable to the human eye (34). Wavelet transformations conduct filtration and noise removal to the original images. Thus, these transformed features may effectively capture critical tumor heterogeneity and better predict tumor biology. The wavelet features may reflect certain cytological characteristics or specific expressions of certain molecules of the tumor microenvironment (35). The underlying biological mechanism remains to be elucidated. When focusing on the features from peritumoral 10-mm regions, the wavelet features tend to show higher expressions in LMPA (5.821 ± 1.21), followed by GBM (5.628 ± 1.03), and META (5.356 ± 0.75). As one of the most common types of intracranial tumors, META is well demarcated from the brain parenchyma. The peritumoral of META dominantly manifests as vasogenic edema and the absence of tumor cells (36). Thus, compared with the features within the intratumoral regions, the features from PBZ regions may also reflect the biological characteristics of the tumor from the perspective of peritumoral infiltration. Similarly, like GBM, LMPA also diffusely infiltrates peritumoral brain tissue adjacent to enhanced tumor masses (37). The high expression of the textural feature around the LMPA improves the prediction performance of the binary classification model (**Figures 4B, C**). The microscopic tumor infiltration of LMPA is reportedly indistinguishable from vasogenic edema or even from normal brain tissue on T2 images (38). This may explain why the textural feature from peritumoral 10-mm regions of LMPA patients was significantly higher than that in META patients. Our results can be further supported by a previous MRI study (39) which showed that increased peritumoral perfusion indicators in LMPA and GBM can reveal tumor-related changes beyond the enhancing borders of the solid tumor entities. Additionally, past research has shown that the apparent diffusion coefficient (ADC) map can provide additional information for the differential diagnosis of malignant brain tumors (40, 41). Zhang et al. (41) reported that the ADC-based texture analysis can help differentiate GBM from solitary brain metastasis. Choi et al. (40) found that ADC parameters extracted from the tumor were higher in GBM than that in LMPA. These findings suggest that the diagnostic performance of our model might be further improved in the future by combining quantitative features around the tumor in images from different modalities (such as ADC). Taken together, our results show that quantitative radiomic features provide a potentially useful complement to the noninvasive assessment of a CNS tumor and may provide imaging guidance for future individualized oncology.

There are several limitations to the present study. First, although multi-center data were included in this research, the overall sample size is still relatively small. Prospective studies carried out at multi-centers with larger sample sizes are required to confirm these findings. Second, our radiomic analysis only used T1CE and T2 images, which are the most common structural MR images in the clinical context. In fact, GBMs bear genomic and imaging heterogeneity, either in intratumoral or peritumoral regions (7, 17). In future research, additional imaging techniques, such as dynamic susceptibility contrast imaging or functional MRI technique, are needed to reveal the underlying landscape of aggressiveness of the tumor.

In conclusion, by incorporating peritumoral information into the model, the presented classifier can differentiate GBM from LMPA and META preoperatively. We believe that the combination of peritumoral information might help to further improve the preoperative evaluation of CNS tumors and guide clinical practice.

## Data availability statement

The original contributions presented in the study are included in the article/**Supplementary Material**. Further inquiries can be directed to the corresponding authors.

## Ethics statement

The studies involving human participants were reviewed and approved by the Institutional Ethical Committee for Clinical Research of the Affiliated Brain Hospital of Nanjing Medical University. The patients/participants provided their written informed consent to participate in this study. Written informed consent was obtained from the individual(s) for the publication of any potentially identifiable images or data included in this article.

## Author contributions

Study design: HL and WZ. Data acquisition: DL, HG, GH, KY, BL, CX, and ZY. Data analysis: DL, JC, XH, and YL. Drafting the article: DL. Pathological analysis: KS. Study supervision: HL, WZ, and YZ. All authors contributed to the article and approved the submitted version.

## Funding

This study was supported by grants from the National Natural Science Foundation of China (No. 81972350, 81701671), the Postgraduate Research & Practice Innovation Program of Jiangsu Province (No. SJCX21_0643), the project of Jiangsu Provincial Medical Youth Talent (No. QNRC2016047), the Medical Scientific and Technologic Development Project of Nanjing (No. ZKX15035, No. YKK20097), the Jiangsu Provincial Medical Innovation Team (No. CXTDA2017050).

## Acknowledgments

This study was supported by grants from the National Natural Science Foundation of China (nos. 81972350 and 81701671), the Postgraduate Research & Practice Innovation Program of Jiangsu Province (no. SJCX21_0643), the project of Jiangsu Provincial Medical Youth Talent (no. QNRC2016047), the Medical Scientific and Technologic Development Project of Nanjing (no. ZKX15035 and no. YKK20097), and the Jiangsu Provincial Medical Innovation Team (no. CXTDA2017050). We express our gratitude to Dr. Ni Hongbin, Chen Weitao, and Liu Hao for their assistance during the data collection.

## Conflict of interest

The authors declare that the research was conducted in the absence of any commercial or financial relationships that could be construed as a potential conflict of interest.

## Publisher’s note

All claims expressed in this article are solely those of the authors and do not necessarily represent those of their affiliated organizations, or those of the publisher, the editors and the reviewers. Any product that may be evaluated in this article, or claim that may be made by its manufacturer, is not guaranteed or endorsed by the publisher.

## Abbreviations

Adaboost, adaptive boosting; AI, artificial intelligence; AUC, area under the receiver-operating-characteristic curves; CE-MRI, contrast-enhanced MRI, CNS, central nervous system; GBM, glioblastoma; GBDT, gradient boosting decision tree; ICCs, the intraclass correlation coefficients; LASSO, least absolute shrinkage and selection operator; LMPA, primary central nervous system lymphoma; META, metastases; MRI, magnetic resonance imaging; NBH, the Affiliated Brain Hospital of Nanjing Medical University; PBZ, peritumoral brain zone; RF, random forest; ROI, regions of interest; SVM, support vector machine; T1CE, T1 contrast-enhanced MRI sequences; T2, T2-weighted MRI sequences.
