# Supplementary material for: Differentiation of malignant brain tumor types using intratumoral and peritumoral radiomic features
Source: Front Oncol. 2022 Jul 28;12:848846. doi: 10.3389/fonc.2022.848846 (PMC9366472; doi:10.3389/fonc.2022.848846)
Supplement: Supplementary file 1 [file DataSheet_1.docx]

**Supplementary Materials for**

**Differentiation of Malignant Brain Tumors Using Intratumoral**

**and Peritumoral Radiomics Features**

**Content:**

[**S1. Multicenter MRI Sequence Scanning Parameters 2**](#_Toc104912499)

[**S2. Image preprocessing and quality control 3**](#_Toc104912500)

[**S3. Region-of-interest segmentation 4**](#_Toc104912501)

[**S4. Radiomics features 5**](#_Toc104912502)

[**4.1 Extraction of the Radiomics features 5**](#_Toc104912503)

[**4.2 Inter-observer stability assessment of features 5**](#_Toc104912504)

[**S5. Research design and classifier selection 6**](#_Toc104912505)

[**S6. Feature Importance And Classifier Assessment 7**](#_Toc104912506)

[**S6.1 LASSO and SVM 7**](#_Toc104912507)

[**S6.2 Other feature selection methods and classifiers 8**](#_Toc104912508)

[**S6.3. The ROC curves and corresponding features of the optimal Adaboost model. 9**](#_Toc104912509)

[**S7. Quantitative features training by Adaboost. 11**](#_Toc104912510)

[**The statistics of the best features from all 5854 features used in Adaboost. 12**](#_Toc104912511)

[**S8. The results for binary classification 12**](#_Toc104912512)

[**S8.1 Binary classification between GBM and LMPA 12**](#_Toc104912513)

[**S8.2 Binary classification between GBM and META 13**](#_Toc104912514)

[**S8.3 Binary classification between LMPA and META 14**](#_Toc104912515)

**Abbreviation**

Adaboost, adaptive Boosting; AUC, area under the receiver-operating-characteristic curves; GBM, glioblastoma; GBDT, gradient boosting decision tree; IBSI, the Image Biomarker Standardization Initiative; ICCs, the intraclass correlation coefficients; LASSO, least absolute shrinkage and selection operator; LMPA, primary central nervous system lymphoma; META, metastases; MRI, magnetic resonance imaging; NBH, the Affiliated Brain Hospital of Nanjing Medical University; NDTH, the Nanjing Drum Tower Hospital. PBZ, peritumoral brain zone; RF, random forest; ROI, regions of interest; SVM, support vector machine; T1CE, T1 contrast-enhanced MRI sequences; T2, T2-weighted MRI sequences; TCIA, The Cancer Imaging Archive;

# S1. Multicenter MRI Sequence Scanning Parameters

Data information of some GBM patients is provided on the website of The Cancer Imaging Archive (**TCIA**, https://wiki.cancerimagingarchive.net/display/Public/TCGA-GBM). MRI images from the Nanjing Brain Hospital-Brain Tumor Neuroimaging Project (NBH-BTnp) database were acquired by 3.0 Tesla MR scanner (Verio, Siemens) in the department of radiology, **NBH.** NBH-BTnp is derived from a Brain Tumor Neuroimaging Project that was launched in January 2013 by Department of Neurosurgery, the Affiliated Brain Hospital of Nanjing Medical University, and was updated in September 2017 by the Institute of Brain Functional Imaging, the Affiliated Brain Hospital of Nanjing Medical University. The goal of NBH-BTnp is to identify early neuroimaging biomarkers of tumors (i.e., Glioma and Meningioma) to protect against the detrimental effects of tumors invading cogntive function, to predict surgical prognosis, recurrence, radiotherapeutic and chemotherapeutic efficacy, to understand compensatory mechanisms to improve comprehensive preoperative planning and the development of neurorehabilitation strategies in patients. All subjects in NBH-BTnp were recruited initially from inpatients and outpatients. This database used a standardized clinical evaluation protocol that included a medical history interview, neurologic examination, a battery of neurocognitive assessment, and multiparameter MRI scans for the participants (healthy controls, glioma, and meningioma). All subjects and their study partners completed the informed consent process, and the study protocols were reviewed and approved by the responsible Human Participants Ethics Committee of the Affiliated Brain Hospital of the Affiliated Brain Hospital of Nanjing Medical University. Additionally, MRI images in test set were acquired by 3.0 Tesla MR scanner (Achieva, Philips) in the department of radiology in the Nanjing Drum Tower Hospital (**NDTH)**.

Sequence parameters for T2, post-contrast T1 (T1CE) acquisition were as follow:

The parameters for T1CE in **NBH** dataset are listed as follows: repeat time (TR)= 2300 ms, echo time (TE)= 2.32-2.82 ms, matrix = 256 x256, flip angle (FA)= 70-90, thickness =0.9-3.0 mm, echo train length = 1-10, pixel bandwidth = 200, spacing between slices = 1-6 mm.

The parameters for T2 in **NBH** dataset are listed as follows: repeat time (TR)= 3500-3800 ms, echo time (TE)= 104-117 ms, matrix = 384 x 384, flip angle (FA)= 90-150, thickness = 1-6 mm, echo train length = 18-26, pixel bandwidth=219-295, spacing between slices = 5-7.8mm.

The parameters for T1CE in **NDTH** dataset are listed as follows: repeat time (TR)= 1500 ms, echo time (TE)= 369 ms, matrix = 256 x220, flip angle (FA)= 56, thickness =1 mm, echo train length = 180, pixel bandwidth = 520, spacing between slices = 1-5 mm.

The parameters for T2 in **NDTH** dataset are listed as follows: repeat time (TR)= 1500-2332 ms, echo time (TE)= 80-369 ms, matrix = 256 x 272, flip angle (FA)= 56, thickness = 1-6 mm, echo train length = 180, pixel bandwidth=212-520 , spacing between slices = 1-7mm.

The training group consisting of T1CE and T2 GBM images was also included from the Cancer Imaging Archive (<http://www.cancerimagingarchive.net>).

The parameters for **3D GRE** protocol in **TCIA** dataset are listed as follows:

**T1CE**: Repeat time (TR) = 25-35 ms, echo time (TE) = 3.7-4.2 ms, matrix = 256-300x192-300, flip angle (FA) =8-15, thickness = 0.9-1 mm, echo train length = 0-1, pixel bandwidth = 180-216, spacing between slices = 1 mm.

The parameters for **2D SE** protocol in **TCIA** dataset are listed as follows:

**T1CE**: Repeat time (TR)= 500-850 ms, echo time (TE)= 9-14 ms, matrix = 192x256x192-256, flip angle (FA)= 90, thickness = 5 mm, echo train length = 0-1, pixel bandwidth = 81-125, spacing between slices = 6.5-7.5 mm.

The parameters for **T2** in **TCIA** dataset are listed as follows: repeat time (TR)= 552-1500 ms, echo time (TE)= 25-98 ms, matrix = 174-244 x 256-320, flip angle (FA)=20-90, thickness = 3-5 mm, echo train length =1-8, pixel bandwidth= 80-122, spacing between slices = 4-6.5mm.

# S2. Image preprocessing and quality control

All MRI images are visually inspected to exclude images with distortions or quality problems (n = 9) before preprocessing. Firstly, all MRI protocols are resampled to 1*1*1 mm voxel resolution. To weaken the effect of voxel interpolation on the further image feature calculation, we used a conservative trilinear interpolation algorithm recommended by the Image Biomarker Standardization Initiative (IBSI)[^1^](#_ENREF_1). Then, a non-linear registration was applied to match the resampled T2 data to the individualized T1CE using SPM12 (https://www.fil.ion.ucl.ac.uk/spm/software/spm12/) toolbox implemented in MATLAB. Next, the registered images were skull-stripped to remove background noise and nonbrain tissue using the brain extraction tool (BET) function in FMRIB software library (FSL version 6.0, <https://fsl.fmrib.ox.ac.uk/fsl/fslwiki/>). Subsequently, the images were subjected to N4 bias correction to remove the bias field artifacts from the images. After bias correction, signal intensities were also normalized using the WhiteStripe normalization packages[^2^](#_ENREF_2). Finally, a low-level non-linear filtering named smallest univalue segment assimilating nucleus (SUSAN) in FSL [^3^](#_ENREF_3) was performed, which can reduce high-frequency noise without blurring the MR images. The registration as well as the following processed steps were examined by visual inspection to ensure the quality of the image data. The histograms density curves of the raw MRI and the preprocessed signal intensities are plotted for examination.

During data preprocessing, patients were excluded from the study due to misregistrations (n = 3) or images standardization problems of either T1CE or T2 images (n = 11). Images that fulfilled the predefined criteria were used as source data for further analysis and intensity curve plotting. The histograms of signal intensities are shown below. Each curve represents the histogram’s distribution of the MRI sequence of a single subject. Figure **S1A** and Figure **S1C** (left column) describe the signal heterogeneity of raw T1CE and T2 images across all included patients (n=250) before N4 bias-correction and intensity normalization. The right column (Figure **S1B** and **S1D**) shows the intensity curves of corrected T2 and T1CE images, respectively.

**Figure S1**


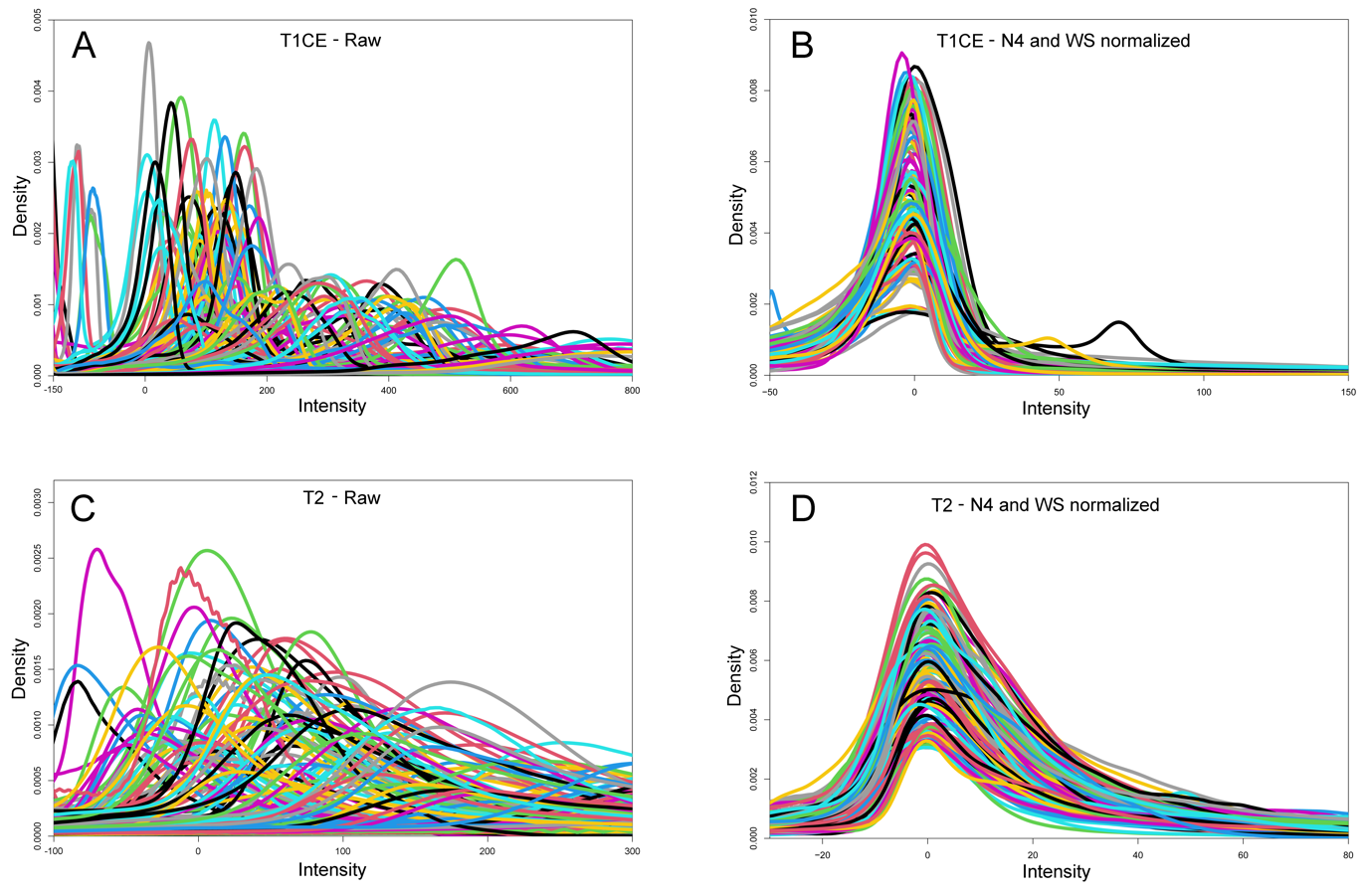


# S3. Region-of-interest segmentation

In our study, the traditional subregions refer to ROIs visible to the naked eye on the different MRI sequences, including enhancement and whole tumor parts in T1CE as well as the edema in T2 sequences. The PBZ subregions start from the boundaries of the enhanced lesion in the T1CE sequence and are quantitatively defined by a certain spatial distance, such as 10mm, 20mm, and 30 mm.

For the visible tumor portions, the outline of the tumors was delineated through a semi-automatic threshold and seed-growing algorithm (Active Contour Segmentation Mode) in ITK-SNAP software (<http://www.itksnap.org/pmwiki/pmwiki.php>). The ROIs defined by this method are highly effective and repeatable. Similarly, this approach was used to segment individualized brain mask without ventricle, sulus, and subtentorial structures. Semi-automated ROI delineation was performed independently by two neuroradiologists that blinded to the clinical data (one with 11 years experience and the other with 7 years experience), following by the expansion calculations. The morphological binary expansion of the whole-tumor is performed to delineate the PBZ regions, which are confined within the individualized whole-brain mask. Briefly, in each iteration of isotropic dilation, a 1 mm^3^ ring around the enhanced lesions will be obtained and the iterations eventually reach a radial distance of 30 mm. Then, the tumor masks were subtracted from the expanded mask to get the distance quantitative PBZ ROIs. Those ROIs were automatically extracted using Python script. Finally, all intratumoral and peritumoral ROIs of each subject were visually examined and modified by the two neuroradiologists independently.

# S4. Radiomics features

## 4.1 Extraction of the Radiomics features

The extracted radiomics features included first-order statistical, shape features,  intensity-histogram-based features, and texture matrix-based features from both unfiltered and filtered images (wavelet decompositions). Texture features contain multiple categories, including gray-level co-occurrence matrix (GLCM), gray-level run-length matrix (GLRLM), gray-level size zone matrix (GLSZM), and gray-level dependence matrix (GLDM), as well as neighborhood gray-tone difference matrix (NGTDM). The details of the features are available on the [official](javascript:;) [website](javascript:;) of pyradiomics[^4^](#_ENREF_4) (<https://pyradiomics.readthedocs.io/en/latest/features.html>).

For each patient, the tumor habitat contains both visible and invisible regions and will be divided into ten subregions (five in T1CE sequence and five in T2 sequence). The ROIs from T1CE including two visible regions (contrast-enhanced tumor, whole tumor) and three invisible regions (peritumoral 10/20/30 mm ROIs (P1/P2/P3) in peritumoral regions). Likewise, the ROIs from T2 including one visible regions edema (ED) and four invisible regions (whole tumor ROI from T1CE and P1/P2/P3). We did not include the necrosis subregions because many tumors may not be accompanied by obvious necrosis (especially in lymphoma and partial metastatic tumor cases).

For each visible subregion ROI, a total of 851 features were extracted, including 14 shape, 18 first-order, 75 texture-related features (24 GLCM, 16 GLRLM, 16 GLSZM, 14 GLDM, 5 NGTDM), and 744 wavelet features from the filtered images. For each invisible subregion ROI, a total of 837 features were extracted in the same way (851-14, except for 14 shape features). Finally, a total of 8412 features (851*3 + 837*7) were obtained for each tumor.

## 4.2 Inter-observer stability assessment of features

According to methods used by IBSI[^1^](#_ENREF_1), the reproducibility of the features was assessed using a two-way, random-effects, single-rater, absolute agreement intraclass correlation coefficient (ICC) with a confidence level of 95% using the ‘irr’ (version 0.84.1) package in R. Admittedly, ROIs derived from different obsevers may lead to inter-observer variability, and potential decreased reproducibility. To ensure the repeatability of the features for further modeling, we used relatively strict criteria for stability evaluation. As suggested by Koo and Li[^5^](#_ENREF_5), we selected the features with ICC values greater

than 0.90 (excellent reliability) for further analysis. **Figure S2** shows the Boxplot of ICCs of the features extracted from four feature categories.

**Figure S2**


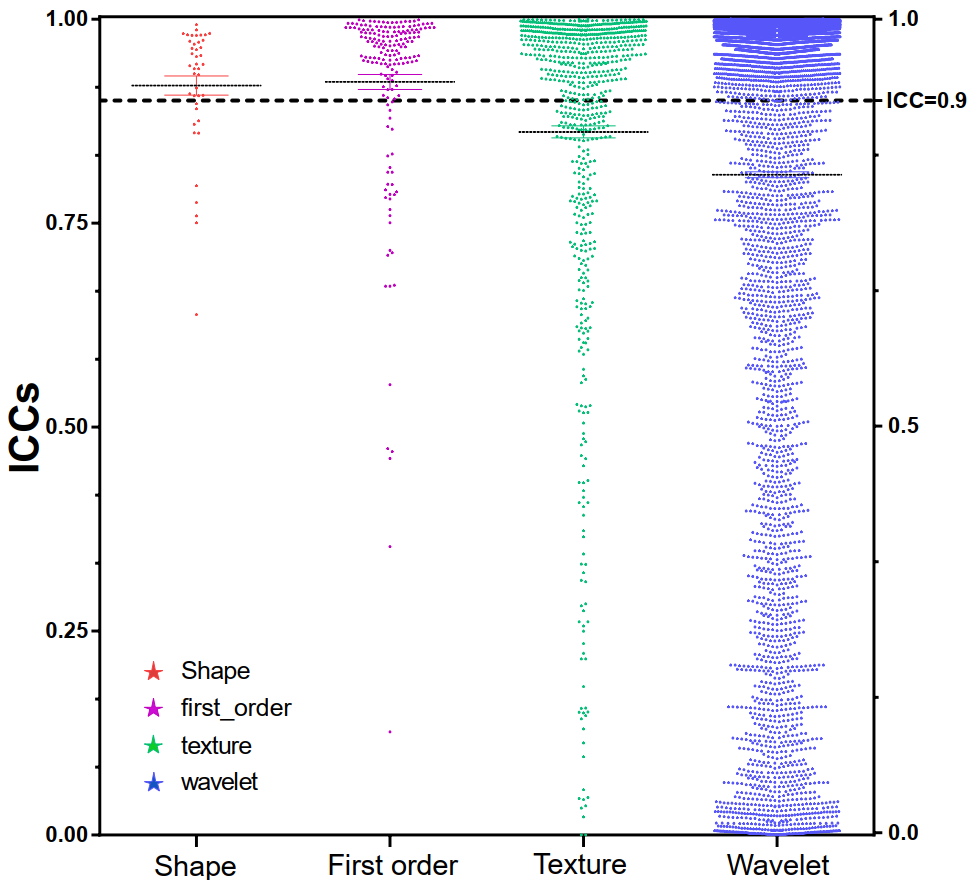


Legend: Each point represents a radiomics feature and different colors represent different feature categories. The points above the thick dotted line are the features with ICCs>0.9.

# S5. Research design and classifier selection

To make the model selection more objective, we first select the best classifier based on traditional features (from intratumoral and edema regions). Based on the top 20 features, the optimal model will be used for three-class differential diagnosis (**Step 1**). Similarly, the same approach will be used for three-classification using quantitative features (features from peritumoral and nonvisual regions) (**Step 2**). Furthermore, using the classifier determine by Step 1, all features will be put together to identify the top 20 features according to the importance of the feature, which will also be used for three-classification (**Step 3**). At last, using top features identified in Step 3, binary classification will be performed through the optimal classifier (**Step 4**).

**Figure S3**


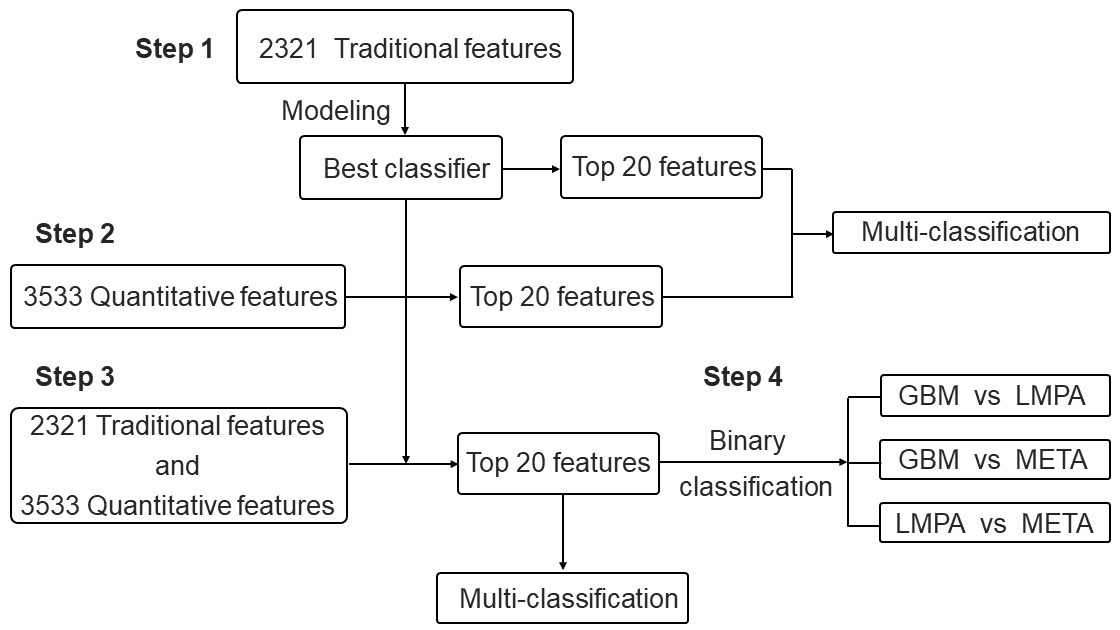


# S6. Feature Importance And Classifier Assessment

## S6.1 LASSO and SVM

After feature stability evaluation by ICC, 2321 features were selected for LASSO. Also, 10-fold cross-validation was used in order to optimize hyper-parameter configurations and to select the best performing configuration. The final top 20 features including 1 texture feature and 19 wavelet features. The Lasso path and MSE (mean squared error) in **Figure S4** describes the relationship between the regression coefficients of the independent variable and the Lasso penalty coefficient. The coefficients of Lasso are listed in **Table S1.**

**Figure S4**


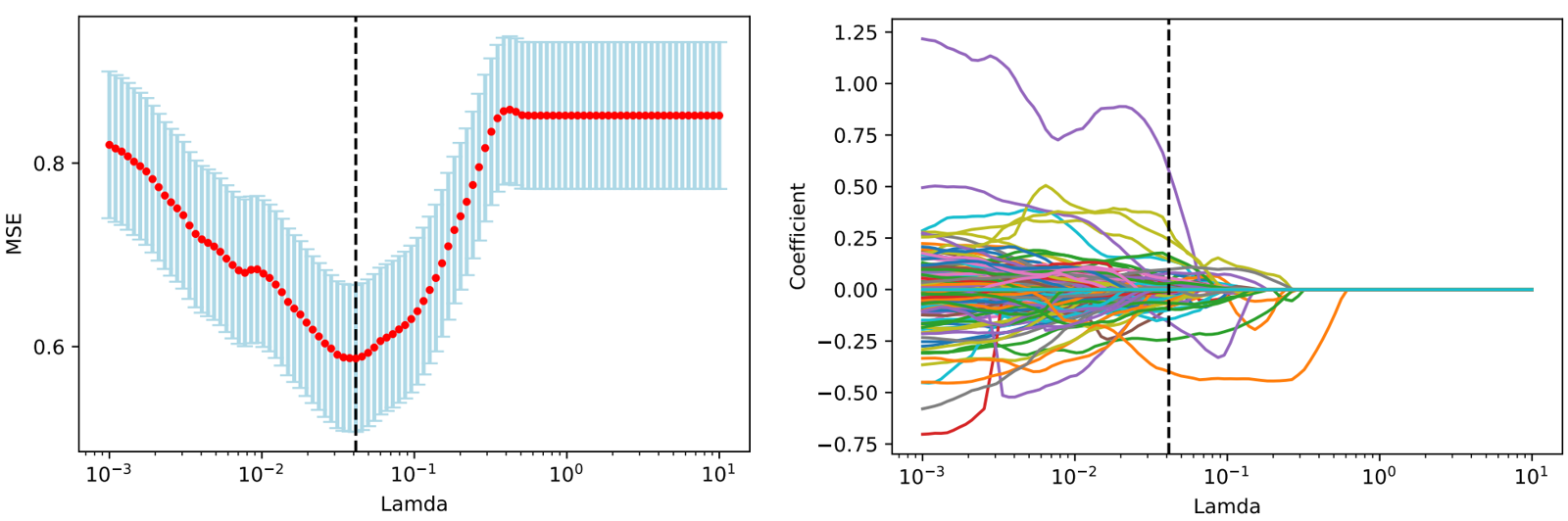


**Table S1**

| **Index** | **Feature name** | **Lasso coefficients** |
| --- | --- | --- |
| **1** | T2_BN4WSSU_ED_wavelet-LHH_glrlm_RunLengthNonUniformityNormalized | 0.175058 |
| **2** | T2_BN4WSSU_ED_wavelet-LLH_firstorder_Mean | 0.131525 |
| **3** | T1CE_BN4WSSU_WT_original_glcm_InverseVariance | 0.1116 |
| **4** | T2_BN4WSSU_ED_wavelet-LHH_glszm_SmallAreaLowGrayLevelEmphasis | 0.10733 |
| **5** | T2_BN4WSSU_ED_wavelet-HHL_glcm_ClusterShade | 0.098433 |
| **6** | T1CE_BN4WSSU_WT_wavelet-HLH_firstorder_Skewness | 0.090534 |
| **7** | T1CE_BN4WSSU_WT_wavelet-LLL_ngtdm_Strength | 0.07412 |
| **8** | T1CE_BN4WSSU_CT_wavelet-LHL_glcm_Idn | 0.074022 |
| **9** | T2_BN4WSSU_ED_wavelet-LHH_glcm_Correlation | 0.06474 |
| **10** | T2_BN4WSSU_ED_wavelet-LLL_glrlm_LongRunHighGrayLevelEmphasis | 0.064612 |
| **11** | T1CE_BN4WSSU_WT_wavelet-LHL_glszm_SizeZoneNonUniformityNormalized | 0.057854 |
| **12** | T1CE_BN4WSSU_CT_wavelet-HHH_glcm_Imc2 | 0.054444 |
| **13** | T1CE_BN4WSSU_WT_wavelet-HHH_glszm_LargeAreaHighGrayLevelEmphasis | 0.05338 |
| **14** | T1CE_BN4WSSU_WT_wavelet-HHH_glszm_GrayLevelVariance | 0.05153 |
| **15** | T1CE_BN4WSSU_WT_wavelet-HHL_gldm_DependenceNonUniformityNormalized | 0.05047 |
| **16** | T1CE_BN4WSSU_WT_original_glrlm_RunLengthNonUniformity | 0.04593 |
| **17** | T1CE_BN4WSSU_CT_wavelet-HLL_glcm_JointAverage | 0.04187 |
| **18** | T2_BN4WSSU_ED_wavelet-LLL_firstorder_Skewness | 0.04183 |
| **19** | T2_BN4WSSU_ED_wavelet-HHL_firstorder_Mean | 0.04122 |
| **20** | T2_BN4WSSU_ED_wavelet-HLL_glrlm_RunVariance | 0.040771 |

Based on the features selected by lasso, the SVM classifier only achieves an AUC of 0.5929 and 0.5996, respectively.

## S6.2 Other feature selection methods and classifiers

The performances of various models are listed in **Table S2.** Compared with SVM, Random Forest, GBDT, and ExtraTree, the Adaboost classification model performs better in terms of all metrics.

**Table S2**

| **Model** | **AUC** | **Precision** | **Recall** | **F1_score** |
| --- | --- | --- | --- | --- |
| **LASSO-SVM(linear)** | 0.5929 | 0.4809 | 0.48 | 0.4174 |
| **LASSO-SVM(rbf)** | 0.5996 | 0.4965 | 0.48 | 0.4343 |
| **Random forest** | 0.6181 | 0.4463 | 0.44 | 0.4191 |
| **Adaboost** | 0.7926 | 0.7066 | 0.64 | 0.6393 |
| **GBDT** | 0.7419 | 0.6543 | 0.56 | 0.5184 |
| **ExtraTree** | 0.6383 | 0.4652 | 0.44 | 0.3303 |

**Note:** Six classifiers (Lasso_SVM (rbf and linear), Adaboost, RF, GBDT, and ExtraTree) were trained by training data and then applied to the independent test set to evaluate the performance. All results were obtained through 10 cross-validations.

## S6.3. The ROC curves and corresponding features of the optimal Adaboost model.

**Figure S5** shows the ROC curves of the best Adaboost model for the differentiation of GBM, META, and LMPA (using the top 20 features of the 2321 traditional regions). The model achieved an average AUC of 0.82 and the AUC of 0.85, 0.88, 0.69 for GBM, LMPA, and META, respectively. **Table S3** summarizes the Importance of the best traditional features used for Adaboost.

**Figure S5**


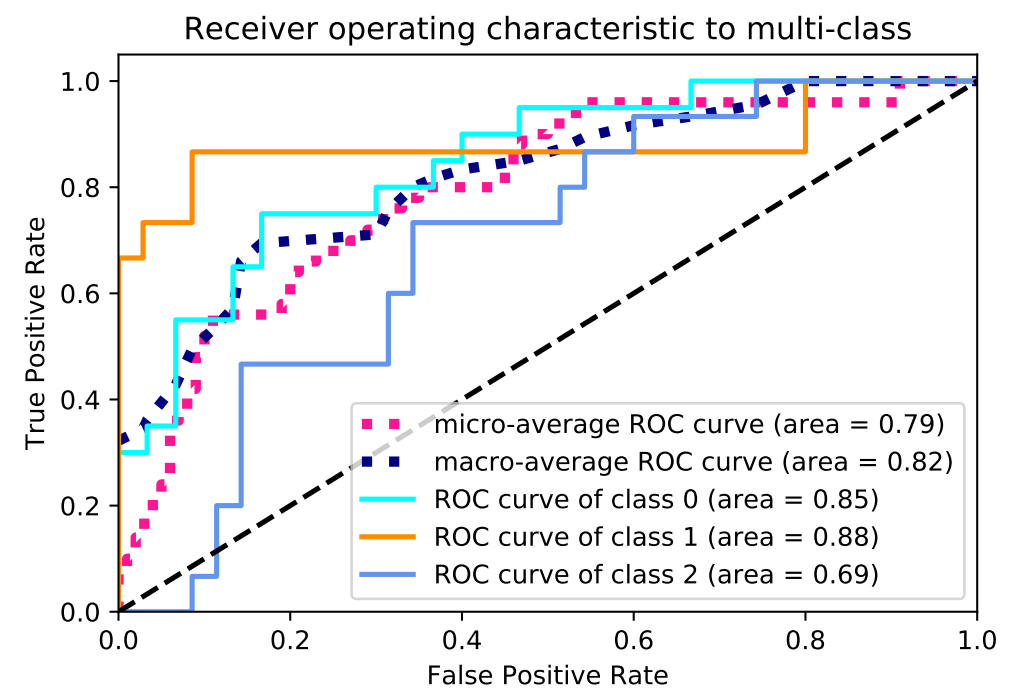


**Legend:** The three ROC curves were averaged into one ROC curve using macro- and micro-averaged methods respectively to get a consensus curve, from which the area under the ROC (AUC) was calculated. Class 0,1,2 refers to GBM, LMPA, and META, respectively. All results were obtained through 10 cross-validations on the test set.

**Table S3**

**Importance of the traditional features for Adaboost in three-classification task**

| **Traditional Feature name** | **Feature importance** |
| --- | --- |
| T1CE_WT_wavelet-HLL_firstorder_Skewness | 0.0858 |
| T1CE_WT_original_glcm_Imc2 | 0.08 |
| T1CE_WT_wavelet-LHL_glcm_MaximumProbability | 0.0752 |
| T1CE_WT_wavelet-HHL_gldm_DependenceNonUniformityNormalized | 0.0744 |
| T1CE_WT_wavelet-HLL_glcm_ClusterShade | 0.0672 |
| T2_ED_wavelet-LHH_glszm_SmallAreaLowGrayLevelEmphasis | 0.0672 |
| T2_ED_wavelet-HHH_firstorder_Mean | 0.064 |
| T2_ED_wavelet-LHL_glszm_GrayLevelNonUniformity | 0.0614 |
| T1CE_CT_wavelet-LLL_glcm_MCC | 0.0554 |
| T2_ED_wavelet-LLL_firstorder_Skewness | 0.0508 |
| T2_ED_wavelet-LHH_glszm_GrayLevelNonUniformity | 0.0438 |
| T2_ED_wavelet-HLL_firstorder_Mean | 0.0382 |
| T2_ED_wavelet-HLH_glszm_GrayLevelNonUniformity | 0.0378 |
| T1CE_WT_wavelet-LLL_ngtdm_Busyness | 0.037 |
| T1CE_CT_wavelet-LLL_glcm_Correlation | 0.0366 |
| T1CE_WT_original_shape_Sphericity | 0.0344 |
| T2_ED_wavelet-HLH_firstorder_Mean | 0.0288 |
| T1CE_CT_original_glcm_Correlation | 0.0222 |
| T2_ED_wavelet-LLH_firstorder_Mean | 0.0208 |
| T1CE_CT_wavelet-LLL_glcm_Imc2 | 0.019 |

**Figure S6**


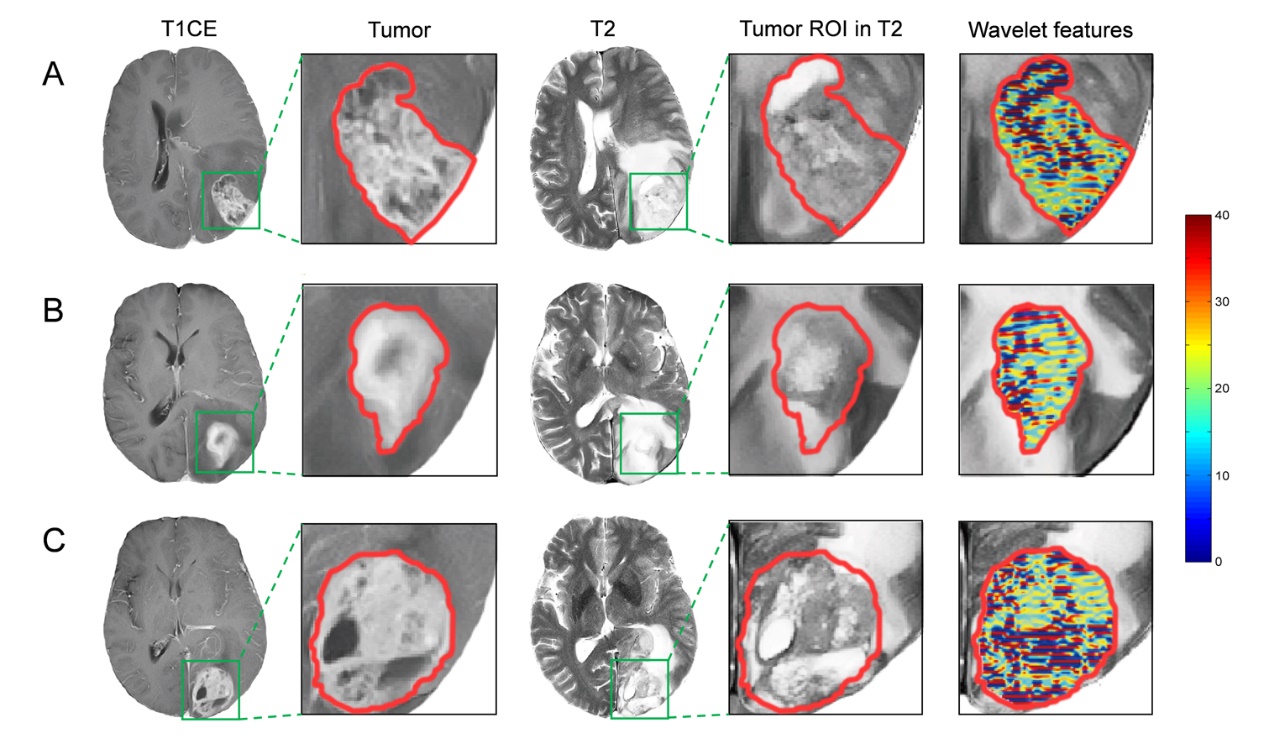


**Legend:**

Each row represents a single patient with a CNS tumor. The first and the second column shows the axial slice of the tumor in T1CE. The third and fourth columns show the edema in the T2 sequence. The ROI of the enhanced tumor that delineated from T1CE was also used for feature extraction in T2. The color heatmap represents the expression of the wavelet feature. **A**, a 49-year-old male with a left temporal-occipital GBM, **B**, a 58-year-old male with a left occipital LMPA; **C**, a 52-year-old male with a left occipital brain metastatic adenocarcinoma.

**Figure S7. The importance of the top 20 features used in the optimal model.**


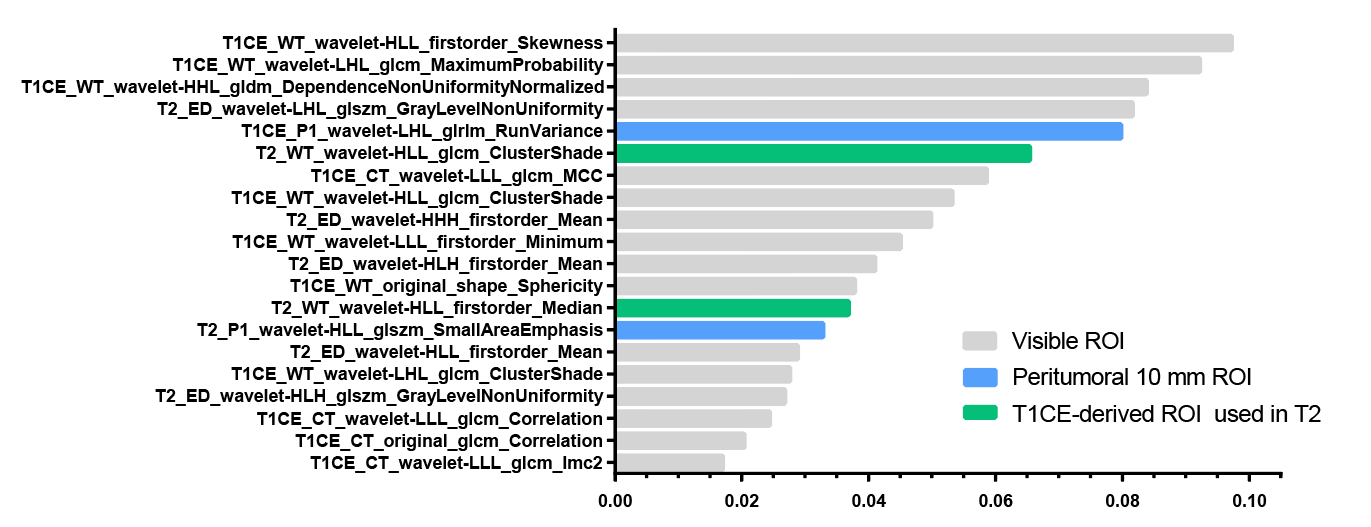


**Legend:** Each bar in the vertical axis represents a radiomics feature and the horizontal axis represents the importance value of the features. The importance value of each feature is calculated and sorted based on the weight in the classification model of the three types of tumors. The grey bars are features from visible regions such as enhancement and edema. The blue bars are features from peritumoral 10 mm regions both in T1CE and T2 images. The green bars represent features from T2 images using T1CE-derived ROI, which are invisible in the original T2-weighted sequence.

# S7. Quantitative features training by Adaboost.

**Figure S8** shows the ROC curve of the best Adaboost model for three-classifying using 3533 quantitative features only. The model achieved an average AUC of 0.77 and the AUC of 0.82, 0.80, 0.66 for GBM, LMPA, and META, respectively.

**Figure S8**


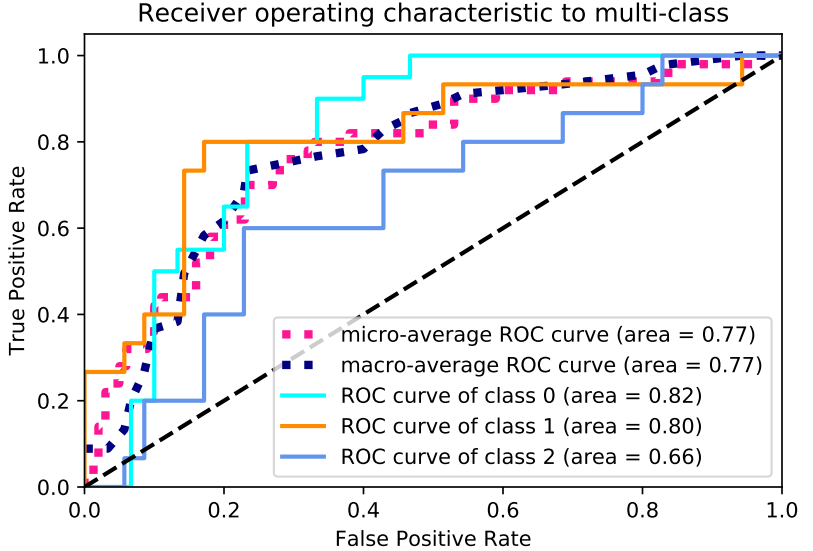


**Legend:** The three ROC curves were averaged into one ROC curve using macro- and micro-averaged methods respectively to get a consensus curve, from which the area under the ROC (AUC) was calculated. Class 0,1,2 refers to GBM, LMPA, and META, respectively. All results were obtained through 10 cross-validations on the test set.

# The statistics of the best features from all 5854 features used in Adaboost.

| **Rank** | **Importance** | **Feature name** | [**Standard**](javascript:;) [**deviation**](javascript:;) | **H value** | **P value** |
| --- | --- | --- | --- | --- | --- |
| 1 | 0.0972 | T1CE_WT_wavelet-HLL_firstorder_Skewness | 0.8850 | 53.119 | 0.000* |
| 2 | 0.0922 | T1CE_WT_wavelet-LHL_glcm_MaximumProbability | 0.0956 | 95.557 | 0.000* |
| 3 | 0.0838 | T1CE_WT_wavelet_HHL_gldm_Dependence_  NonUniformityNormalized | 0.0049 | 25.927 | 0.000* |
| 4 | 0.0816 | T2_ED_wavelet-LHL_glszm_GrayLevelNonUniformity | 28.02 | 14.165 | 0.001* |
| 5 | 0.0798 | T1CE_P1_wavelet-LHL_glrlm_RunVariance | 1.027 | 6.206 | 0.045* |
| 6 | 0.0654 | T2_WT_wavelet-HLL_glcm_ClusterShade | 0.2390 | 71.396 | 0.000* |
| 7 | 0.0586 | T1CE_CT_wavelet-LLL_glcm_MCC | 0.0854 | 53.332 | 0.000* |
| 8 | 0.0532 | T1CE_WT_wavelet-HLL_glcm_ClusterShade | 5.4861 | 77.323 | 0.000* |
| 9 | 0.0498 | T2_ED_wavelet-HHH_firstorder_MeanAbsoluteDeviation | 0.3249 | 11.278 | 0.004* |
| 10 | 0.045 | T1CE_WT_wavelet-LLL_firstorder_Minimum | 127.88 | 72.476 | 0.000* |
| 11 | 0.041 | T2_ED_wavelet-HLH_firstorder_MeanAbsoluteDeviation | 0.5435 | 3.266 | 0.195 |
| 12 | 0.0378 | T1CE_WT_original_shape_Sphericity | 0.0972 | 20.874 | 0.000* |
| 13 | 0.0368 | T2_WT_wavelet-HLL_firstorder_Median | 0.3205 | 28.445 | 0.000* |
| 14 | 0.0328 | T2_P1_wavelet-HLL_glszm_SmallAreaEmphasis | 0.0842 | 2.653 | 0.265 |
| 15 | 0.0288 | T2_ED_wavelet-HLL_firstorder_MeanAbsoluteDeviation | 2.1117 | 2.865 | 0.239 |
| 16 | 0.0276 | T1CE_WT_wavelet-LHL_glcm_ClusterShade | 4.1425 | 71.517 | 0.000* |
| 17 | 0.0268 | T2_ED_wavelet-HLH_glszm_GrayLevelNonUniformity | 1635.8 | 12.831 | 0.002* |
| 18 | 0.0244 | T1CE_CT_wavelet-LLL_glcm_Correlation | 0.0971 | 45.747 | 0.000* |
| 19 | 0.0204 | T1CE_CT_original_glcm_Correlation | 0.1156 | 41.538 | 0.000* |
| 20 | 0.017 | T1CE_CT_wavelet-LLL_glcm_Imc2 | 0.0781 | 56.883 | 0.000* |

Note: The Importance refers to the weight of the features in the adaboost model in the three-classification task. The H and p represent the statistics of the Kruskal-Wallis test among three groups. * p value < 0.05 was considered as significant.

# S8. The results for binary classification

# S8.1 Binary classification between GBM and LMPA

As shown in **Figure S9**, the ROC of the test set contains the independent ROC for each fold, the mean ROC of the 10-fold cross-validation, the mean ROC, and its standard deviation. The model

achieved an average AUC of 0.98 (95% CI: 0.795 to 0.990) for the classification between GBM and LMPA. **Figure S10** illustrates the weights of the features in the model. The importance of the feature from peritumoral 10 mm in T1CE is 0.098.

**Figure S9**


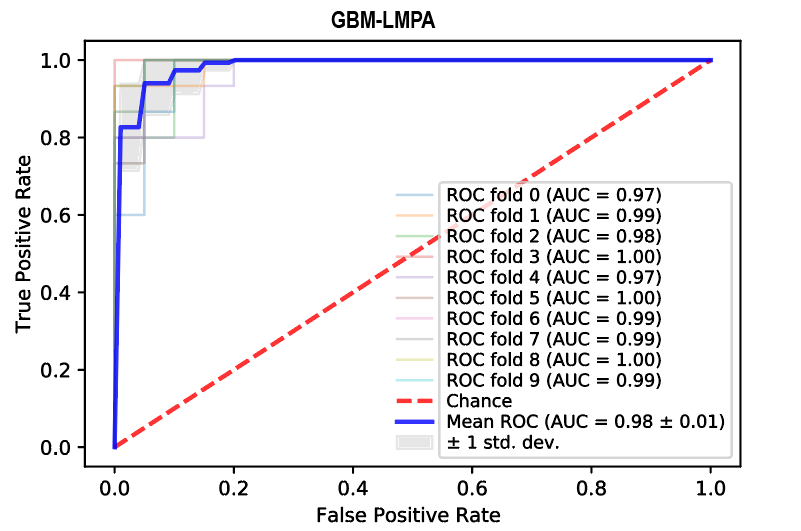


**Figure S10**


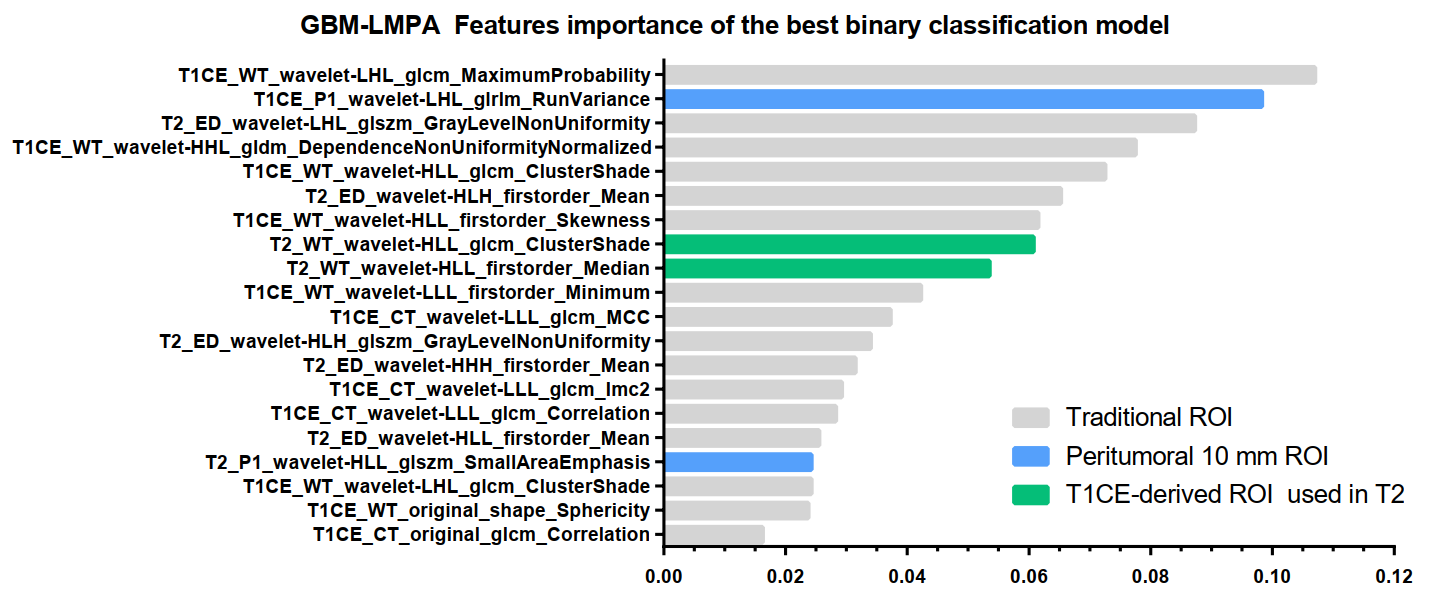


## S8.2 Binary classification between GBM and META

As shown in **Figure S11**, the ROC of the test set contains the independent ROC for each fold, the mean ROC of the 10-fold cross-validation, the mean ROC, and its standard deviation. The model

achieved an average AUC of 0.70 (95% CI: 0.522 to 0.843) for the classification between GBM and LMPA. **Figure S12** illustrates the weights of the features in the model. The importance of the feature from peritumoral 10 mm in T1CE is 0.047.

**Figure S11**


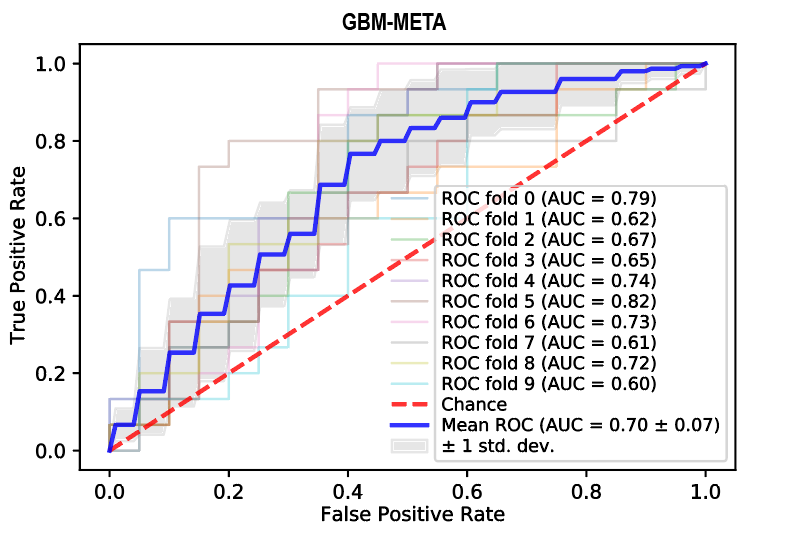


**Figure S12**


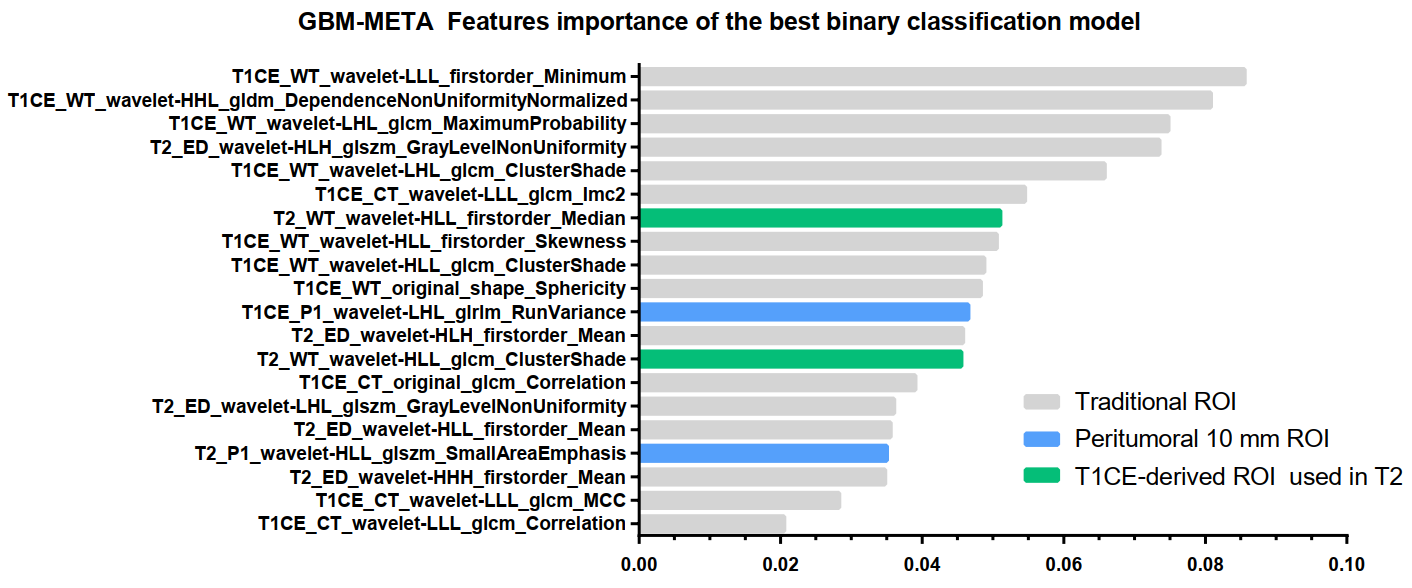


## S8.3 Binary classification between LMPA and META

As shown in **Figure S13**, the model achieved an average AUC of 0.86 (95% CI: 0.614 to 0.923) for the classification between LMPA and META. **Figure S14** illustrates the weights of the features in the model. The importance of the feature from peritumoral 10 mm in T1CE is 0.067.

**Figure S13**


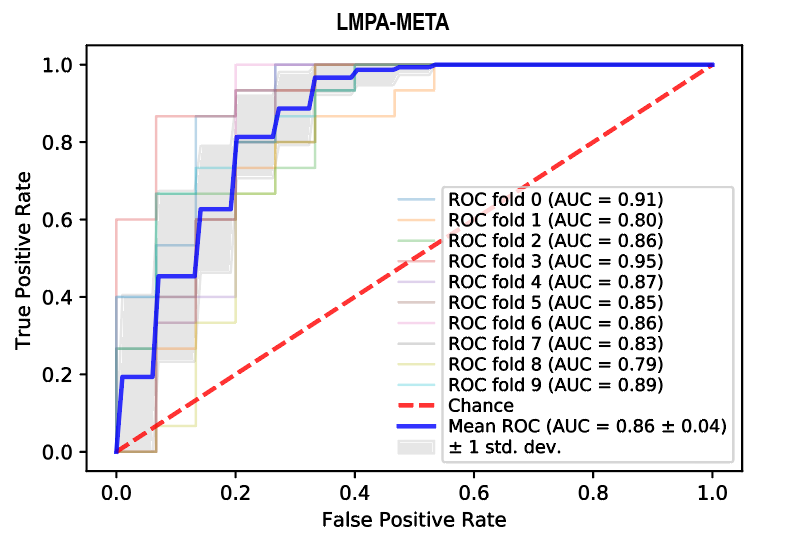


**Figure S14**


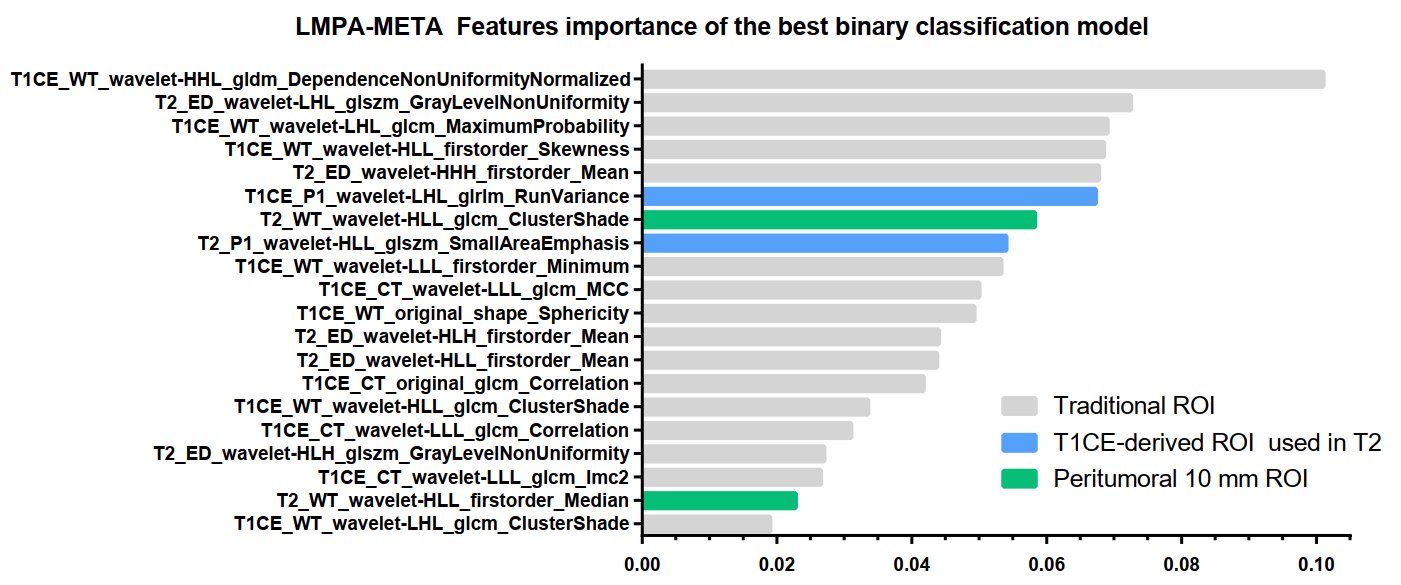


**Reference**

**1.** Zwanenburg A, Vallieres M, Abdalah MA, et al. The Image Biomarker Standardization Initiative: Standardized Quantitative Radiomics for High-Throughput Image-based Phenotyping. *Radiology.* May 2020;295(2):328-338.

**2.** Shinohara RT, Sweeney EM, Goldsmith J, et al. Statistical normalization techniques for magnetic resonance imaging. *NeuroImage. Clinical.* 2014;6:9-19.

**3.** Smith SM, Brady JM. SUSAN - a new approach to low level image processing. *International Journal of Computer Vision.* 1997;23(1):45-78.

**4.** van Griethuysen JJM, Fedorov A, Parmar C, et al. Computational Radiomics System to Decode the Radiographic Phenotype. *Cancer research.* Nov 1 2017;77(21):e104-e107.

**5.** Koo TK, Li MY. A Guideline of Selecting and Reporting Intraclass Correlation Coefficients for Reliability Research. *J Chiropr Med.* Jun 2016;15(2):155-163.
